# Modelling estimates of age-specific influenza-related hospitalisation and mortality in the United Kingdom

**DOI:** 10.1186/s12889-016-3128-4

**Published:** 2016-06-08

**Authors:** Gonçalo Matias, Robert J. Taylor, François Haguinet, Cynthia Schuck-Paim, Roger L. Lustig, Douglas M. Fleming

**Affiliations:** GSK Vaccines, Avenue Fleming 20, Parc de la Noire Epine, Wavre, Belgium; Sage Analytica, 4915 St. Elmo Avenue, Suite 205, Bethesda, MD 20814 USA; Independent Consultant, 9 Dowles Close, Birmingham, B29 4LE UK

**Keywords:** Influenza, Theoretical model, Regression analysis, Mortality, Hospitalisation, Elderly

## Abstract

**Background:**

Influenza is rarely confirmed with laboratory testing and accurate assessment of the overall burden of influenza is difficult. We used statistical modelling methods to generate updated, granular estimates of the number/rate of influenza-attributable hospitalisations and deaths in the United Kingdom. Such data are needed on a continuing basis to inform on cost-benefit analyses of treatment interventions, including vaccination.

**Methods:**

Weekly age specific data on hospital admissions (1997–2009) and on deaths (1997–2009) were obtained from national databases. Virology reports (1996–2009) of influenza and respiratory syncytial virus detections were provided by Public Health England. We used an expanded set of ICD-codes to estimate the burden of illness attributable to influenza which we refer to as ‘respiratory disease broadly defined’. These codes were chosen to optimise the balance between sensitivity and specificity. A multiple linear regression model controlled for respiratory syncytial virus circulation, with stratification by age and the presence of comorbid risk status (conditions associated with severe influenza outcomes).

**Results:**

In the United Kingdom there were 28,516 hospitalisations and 7163 deaths estimated to be attributable to influenza respiratory disease in a mean season, with marked variability between seasons. The highest incidence rates of influenza-attributable hospitalisations and deaths were observed in adults aged 75+ years (252/100,000 and 131/100,000 population, respectively). Influenza B hospitalisations were highest among 5–17 year olds (12/100,000 population). Of all estimated influenza respiratory deaths in 75+ year olds, 50 % occurred out of hospital, and 25 % in 50–64 year olds. Rates of hospitalisations and death due to influenza-attributable respiratory disease were increased in adults identified as at-risk.

**Conclusions:**

Our study points to a substantial but highly variable seasonal influenza burden in all age groups, particularly affecting 75+ year olds. Effective influenza prevention or early intervention with anti-viral treatment in this age group may substantially impact the disease burden and associated healthcare costs. The high burden of influenza B hospitalisation among 5–17 year olds supports current United Kingdom vaccine policy to extend quadrivalent seasonal influenza vaccination to this age group.

**Trial registration:**

ClinicalTrial.gov, NCT01520935

**Electronic supplementary material:**

The online version of this article (doi:10.1186/s12889-016-3128-4) contains supplementary material, which is available to authorized users.

## Background

Annual influenza epidemics results in illness among individuals in all age groups, and large numbers of hospital admissions and deaths [1–3]. Genetic drift of influenza viruses causes frequent changes in the circulating virus strains and limited continuing immunity from 1 year to the next. The circulating virus subtypes in any one season, the relative impact on different age groups, and the timing of epidemic onset are not readily predictable [4]. The World Health Organization makes recommendations on the virus strains to be used in influenza vaccines for the forthcoming season, but mismatches between the chosen strain and the eventual circulating strain may occur.

Prior to 2012, influenza vaccination in the United Kingdom (UK) was recommended for all individuals aged 65+ years, those with comorbid conditions which defined them as at risk of complications of influenza, and all pregnant women. In 2012, the Joint Committee on Vaccination and Immunisation recommended that influenza vaccination be extended to include all children between 2 and 17 years of age, with phased introduction of the programme beginning in 2013 [5].

Between 1966 and 2006 the incidence of influenza-like illness in England and Wales has declined gradually, albeit with peaks during severe epidemics in some years [6]. The cause of the reduction in incidence is not explained, but similar reductions in incidence have been observed for other respiratory diagnoses in the UK and the Netherlands [7, 8]. These fluctuations emphasise the need for regular re-assessment of the impact of influenza and other viruses using contemporary data. Assessment of the burden of illness due to influenza is not straightforward because not all sufferers consult a doctor, many other respiratory viruses produce similar symptoms and the diagnosis is rarely confirmed by laboratory testing. Moreover, much of the burden results from complications which are not necessarily directly attributed to influenza as the underlying cause.

Statistical modelling techniques are used to explore the total burden of influenza. A widely practiced method measures excess influenza-related health outcomes during the winter over a seasonally variable baseline [9]. However, limited data about other respiratory viruses (including some not yet identified) may lead to overestimation of the influenza burden. Techniques using regression modelling to assign a proportion of an outcome to influenza address these difficulties [2, 10]. Most recently, these methods have been adapted to allow estimation of the proportion of an outcome attributable to a particular pathogen whilst controlling for other pathogens associated with the outcome [11].

We used regression modelling methods to estimate mortality and hospitalisations attributable to influenza in the UK according to age, influenza type and subtype, and considering known comorbid risk factors for severe influenza. We controlled for respiratory syncytial virus (RSV), which has similar winter seasonality to influenza. The results comprehensively describe the burden of hospitalisations and deaths over 12 and 13 seasons, respectively, using several influenza-related outcome definitions of varying sensitivity and specificity. We also report on a new outcome, ‘respiratory disease broadly defined’, that combined all respiratory diagnoses with sepsis, unspecified viral infections and selected presenting symptom. This definition was designed to have high sensitivity while maintaining reasonable specificity.

## Methods

### Study design

We extracted data from national databases in a time series model to estimate the burden of influenza disease in the UK in each season (July in the index year through June in the following year) between 1996 and 2009 (www.clinicaltrials.gov NCT01520935). Seasons after July 2009 were disturbed by the pandemic experience in the summer of 2009 and were not included to allow us to establish the normal or average seasonal burden of influenza.

The inclusion criterion was registration with a potentially influenza-related event (respiratory and all other outcomes) in the Hospital Episode Statistics (HES) or Office of National Statistics (ONS) databases. The protocol was approved by the Independent Scientific Advisory Committee of the Clinical Practice Research Datalink (CPRD). Informed consent was not required.

### Data sources

Weekly virological surveillance data for influenza A, influenza B and RSV were obtained from the UK national surveillance system at Public Health England (PHE). Influenza and RSV reports were based on virus swab-positive nose/throat swabs or nasopharyngeal aspirates notified to PHE. For influenza, strain type A or B was specified on reports but subtype was not always provided, so viruses were classified as A or B.

Data from 1997 to 2009 (data could not be extracted before April 1997), were extracted from the HES database (described in Additional file 1) which captures records from almost all patients admitted to National Health Service non-psychiatric hospitals in England. Records for each influenza-related emergency admission were extracted using International Classification of Diseases (ICD) codes version 10.

All deaths in England and Wales are registered with the ONS (Additional file 1) and coded using ICD classification of cause (ICD-9 prior to 2002, ICD-10 thereafter). Data were extracted from the ONS database from 1996 to 2009. Patients who died in hospital were identified using HES data. As recommended by ONS, we adjusted for this change by applying comparability ratios derived from the average baseline rates of incidence for the respiratory outcomes for which sharp differences (departing from trend estimates) were observed between 2000 and 2001 to the 1996–2000 outcome counts (e.g., 1.22, 1.69 and 2.09 respectively for the primary outcome categories ‘all respiratory diagnoses’, ‘pneumonia and influenza’ and ‘bronchitis and bronchiolitis’). These adjustments produced time series that were not substantially different before and after the version change.

### Outcome definitions

Outcome definitions were designed to span a range of sensitivity and specificity (Additional file 1: Table S1). We developed an outcome category ‘respiratory disease (broadly defined)’ that included codes for diseases of the respiratory system (ICD-10 ‘J’ codes), cough and abnormalities of breathing, non-specific viral infections, and sepsis. A negative control outcome (accidents) was also examined. In this report, unless otherwise noted, all outcomes are based either on the primary diagnosis (hospitalisations and in-hospital deaths) or underlying cause (ONS deaths).

### Comorbid risk status

Each HES admission record and ONS record was reviewed for ICD codes indicative of co-morbidity which would prioritise the patient to receive influenza vaccination [12]: chronic obstructive pulmonary disease, cardiovascular disorders, kidney disorders, diabetes, immunosuppression, liver disorders, stroke, central nervous system disorders (Additional file 1: Table S2). Comorbid risk status could only be based on what was listed on the admission or death record under study, as patients could not be linked to their other health encounters.

### Denominators

The population of the UK (2012) is approximately 63.7 million, of which 53.5 million reside in England, 5.3 million in Scotland, 3.1 million in Wales and 1.8 million in Northern Ireland. We used the population distribution of age-specific risk derived from a companion study set in the CPRD population and extrapolated to the entire UK population in 2001 (ONS, [13]) as denominators for HES and ONS risk-stratified estimates. The companion study established excellent agreement between CPRD and independent data sources for the UK with respect to the population age structure, the prevalence of various risk factors, and influenza vaccination coverage [14].

### Statistical methods

Statistical analyses were performed using SAS 9.2. Weekly time-series of the number of specimens positive for influenza A, influenza B and RSV were generated using PHE virology surveillance data. Weekly time-series for influenza-related health outcomes (Additional file 1: Table S1) were generated from HES and ONS data for age groups <5, 5–17, 18–49, 50–64 (and 65–74 and 75+ examined separately) and low/high comorbid risk group. A multiple linear regression model was applied to each age group to associate hospitalisations or deaths to influenza A or B, while controlling for RSV and unspecified seasonal factors. Major changes in virus detection methodology were undertaken by PHE in 2001; thus, to avoid bias the model separated the periods before and after this date.

β_0_+ β_s1_t + β_s2_t^2^+ β_s3_t^3^+ β_s4_sin(2πt/52.18) + β_s5_cos(2πt/52.18) + β_p1a_Influenza A(pre-July 2001) + β_p1b_Influenza A(post-July 2001) + β_p2a_Influenza B(pre-July 2001) + β_p2b_Influenza B(post-July 2001) + β_p3a_RSV(pre-July 2001) + β_p3a_RSV(post-July 2001)where *t* = time in days since 1 July 1995; Influenza A, Influenza B and RSV are observed counts of positive tests from the PHE LabBase dataset. The influenza burden was derived from parameters βp3a through βp2b.

Pathogen predictor variables were transformed into proportions by dividing weekly positive tests by the total number of positives for the season (July to June). Both predictors produced models with similar explanatory power. Outcome and pathogen series were tri-mean-smoothed. The weekly attribution to each virus was computed as the product of the observed value of the explanatory variable (i.e., number of positive virology samples) and the corresponding regression coefficient (β_p1a_ through β_p2b_), and the weekly estimates summed to produce the seasonal estimates. Confidence intervals were based on the standard error of the pathogen parameter estimate. Specifically, we first took each weekly point estimate, multiplied by the regression coefficient, and aggregated the weekly estimates over the entire season. We then repeated the procedure using the lower and upper 95 % estimates for the regression parameter to obtain the seasonal upper and lower confidence intervals. The variability of annual all-age estimates was assessed using standard deviations. Results were expressed as numbers of hospitalisations or deaths, and as the mean seasonal (from September to mid-May) rates of hospitalisations or deaths per 100,000 population.

We considered the possibility that observed prevalence of pathogens could have its effect on outcomes at a distance of one or more weeks. Therefore, we investigated the effects of lagging the virology data, especially those for RSV because these samples are almost exclusively derived from children <5 years [10]. In the absence of hard data regarding RSV infection in the elderly, we suggest that the efficacy of the lagged variable in the regression models reflects an actual sequential pattern of infection across age groups. The best model fit was obtained by lagging of the RSV viral series by 3 weeks for HES and ONS outcomes in individuals >18 years of age. Lagging the influenza series did not result in consistently higher or lower influenza burden attributions.

The final model was optimised by excluding summer months, smoothing the time series, and consideration of two periods for two pathogen time series (pre- and post-July 2001 season periods corresponding to major changes in virus detection methodology undertaken by PHE at that time).

## Results

### Model fit

The goodness of fit was assessed using adjusted R^2^ (co-efficient of determination). Fit was very good in most strata. For hospitalisations the adjusted R^2^ was greater than 0.87 for the respiratory outcome for all age strata except 5–17 years, which had an R^2^ of 0.51; for deaths, the adjusted R^2^ was above 0.81 for the two oldest age groups, but lower for the younger age groups in which death was a relatively rare outcome. Moreover, we found a substantial “lift” in the adjusted R^2^ value when introducing the virology terms into the base secular model. We did not adjust the model form (by including or excluding terms) individually for each outcome, age, and risk stratum, but applied the given form across all strata. The all-age analyses of influenza-attributable admissions for the control outcome (accidents), disclosed no excesses (Table 1).

**Table 1:** Mean seasonal hospitalisations and deaths (number, rate per 100,000 population, all ages) attributable to influenza

	Influenza A		Influenza B	
Outcome	N (SD)	Rate (SD)	N (SD)	Rate (SD)
Hospitalisations: Primary diagnosis				
Respiratory Broadly defined	27,237 (17,895)	46 (30)	1279 (1914)	2 (3)
Respiratory	25,725 (16,706)	44 (28)	703 (2060)	1 (4)
Pneumonia & influenza	6789 (4269)	12 (7)	236 (1066)	0 (2)
Cardiorespiratory	25,412 (16,818)	43 (29)	57 (2414)	0 (4)
Hospitalisations: Any mention				
Respiratory	28,208 (19,209)	48 (33)	921 (3257)	2 (6)
Accidents (control outcome)	0 (595)	0 (1)	0 (935)	0 (2)
Mortality: Underlying				
Respiratory Broadly defined	6561 (5501)	11 (9)	602 (681)	1 (1)
Respiratory	6478 (5419)	11 (9)	592 (679)	1 (1)
Pneumonia & influenza	3155 (2787)	5 (5)	373 (442)	1 (1)
Cardiorespiratory	11,661 (10,168)	20 (17)	990 (1135)	2 (2)

*N* Number of episodes, *SD* standard deviation, *Rate* per 100,000 population

Hospitalisations from HES 1997–2009, deaths from ONS, 1996–2009

### Hospitalisations

**Fig. 1:**
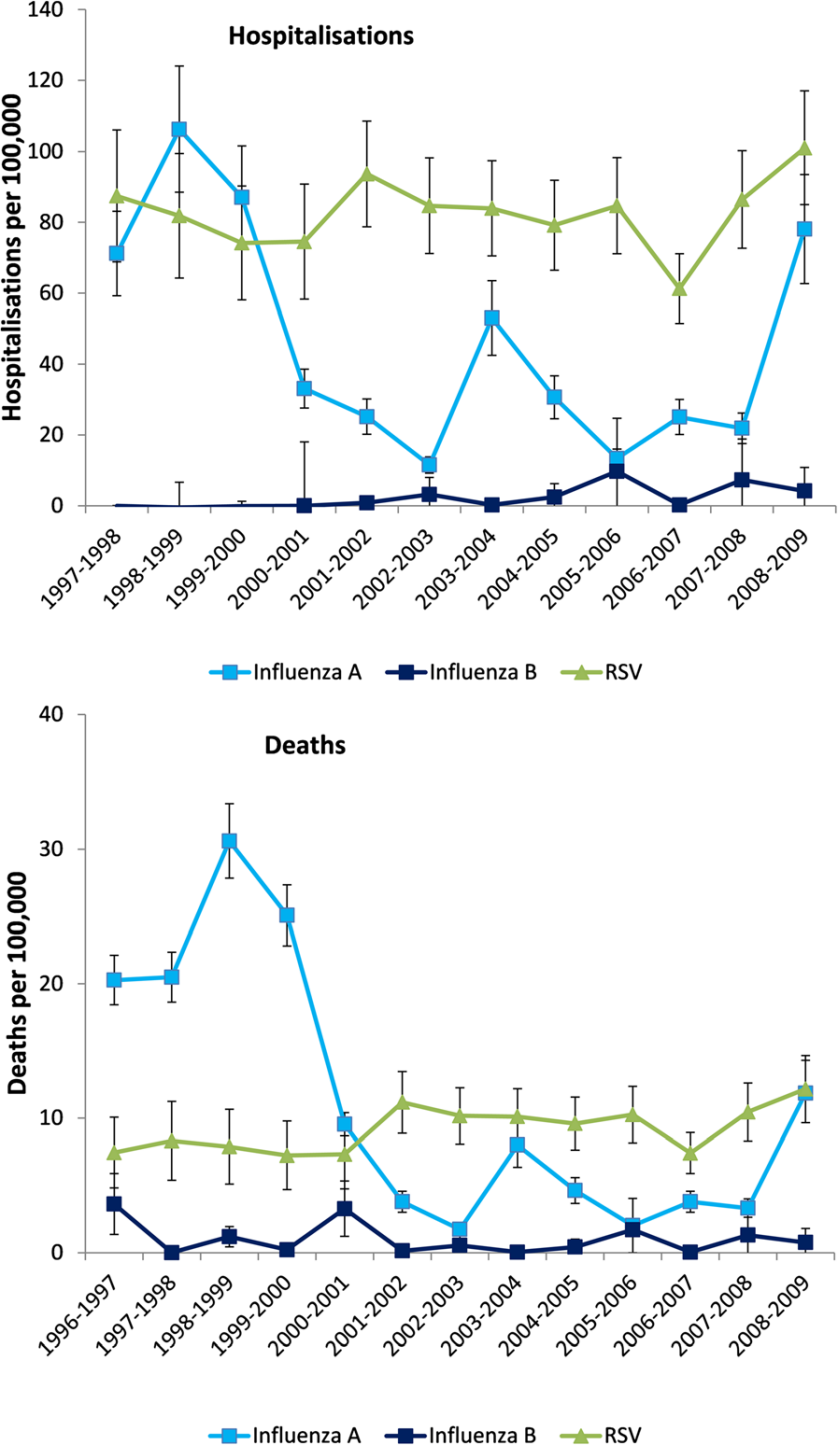
Seasonal hospitalisation and death rates for respiratory disease* attributable to influenza A, B and RSV. Footnote: *vertical bars* = 95 % confidence intervals, *RSV* respiratory syncytial virus *broadly defined

As expected, there was marked variability in influenza-attributable hospitalisation rates due to respiratory disease (broadly defined) from season to season (Fig. 1). In a mean season there were 28,516 hospitalisations for respiratory disease attributable to influenza, corresponding to an overall rate of 49/100,000 population (Table 1). Of these, 27,237 hospitalisations (range across seasons 6757 to 62,427) were attributable to influenza A, and 1279 (range across seasons 0 to 5708) to influenza B, corresponding to an overall rate of 46/100,000 and 2/100,000 population, respectively (Table 1). The all age seasonal estimates of respiratory (primary diagnosis) and cardiorespiratory hospitalisations (primary diagnosis) attributable to influenza were slightly lower than those for respiratory disease (broadly defined).

The highest mean seasonal rates estimate for influenza-attributable hospitalisations were observed in adults 75+ years (Table 2). Our estimate of the influenza-attributable respiratory (broadly defined) hospitalisation rate in this age group was 2-fold higher than that of people 65–74 years, and accounted for 36 % of all hospitalisations.

**Table 2:** Mean seasonal hospitalisations for respiratory disease^a^ attributable to influenza by age and risk status^b^

		Influenza A		Influenza B	
Age	Risk	N (SD)	Rate (SD)	N (SD)	Rate (SD)
0–4	All	3247	93	0	0
	Low	3866	111	0	0
	High	0	0	0	0
5–17	All	796	8	1173	12
	Low	1009	10	1295	13
	High	0	0	144	1
18–49	All	3273	13	652	2
	Low	1754	7	549	2
	High	15,509	59	1501	6
50–64	All	4005	39	51	0
	Low	1454	14	68	1
	High	12,793	124	0	0
65–74	All	5019	101	0	0
	Low	1743	35	0	0
	High	9828	199	0	0
75+	All	10,195	252	0	0
	Low	6625	164	0	0
	High	13,181	326	0	0
All ages^c^	All	27,237 (17,895)	46 (30)	1279 (1914)	2 (3)
	Low	14,238 (9261)	24 (16)	1836 (1803)	3 (3)
	High	84,142 (61,066)	143 (104)	0 (7759)	0 (13)

*N* Number of episodes, *SD* standard deviation, *Rate* per 100,000 population, Hospitalisations from HES 1997–2009

^a^broadly defined

^b^risk group defined according the chronic conditions indicative of risk for severe influenza as per UK recommendations for influenza vaccination. These include chronic obstructive respiratory disease; cardiovascular, central nervous system, renal and liver disorders; diabetes; immunosuppressive conditions or stroke

^c^the attributions for each age group are not equal to the sum of the attributions (all ages) due to separately run models

The mean estimated seasonal hospitalisation rate for respiratory disease attributable to influenza A exceeded those attributable to influenza B for all age groups, except for those aged 5–17 years (Table 2). Influenza B-attributable respiratory admissions exceeded those attributable to influenza A in 5–17 year olds in five of the seasons studied, and in 18–49 year olds in two of the seasons studied (data not shown).

Investigation of non-respiratory diagnoses (cardiovascular disease, central nervous system, diabetes and renal disease) showed a tenuous association between influenza and these outcomes, predominantly in the older age groups (Additional file 1: Table S3).

### Hospital admission mortality rate

Considering mean seasonal influenza-attributed HES deaths as a proportion of all influenza-attributable ONS deaths, 50 % of all respiratory (broadly defined) deaths attributed to influenza occurred in hospital (Table 3). The proportion of all influenza respiratory deaths that occurred in hospital was higher in the 50–64 (75 %) and 65–74 (65 %) year age groups, than in those 75+ years (50 %). The overall mortality rate of influenza-attributable hospitalizations (HES deaths divided by HES hospitalisations) was 12 % for hospitalised patients with a respiratory (broadly defined) diagnosis, and was higher in the elderly (26 % in those aged 75+ years). Over time, there was a trend toward more influenza attributable deaths occurring in hospital (data not shown).

**Table 3:** Hospitalisation and mortality rates due to influenza-attributable respiratory disease^a^ by place of death and age-group

Age group (years)	Hospitalisations (HES)	Deaths (ONS)	Inpatient deaths (HES deaths)	% of hospitalised patients who died in hospital^b^	% of ONS-reported deaths that occurred in hospital^c^
0–4	93	0	0	--	--
5–17	20	0	0	--	--
18–49	15	0	0	--	--
50–64	39	4	3	8 %	75 %
65–74	101	20	13	13 %	65 %
75+	252	131	65	26 %	50 %
All ages	49	12	6	12 %	50 %

Hospitalisations according to primary diagnosis, deaths according to underlying diagnosis

^a^broadly defined

^b^calculated from mean seasonal attributed HES deaths (rate /100,000) divided by mean seasonal attributed hospitalisations (rate /100,000)

^c^calculated from mean seasonal attributed HES deaths (rate /100,000) divided by mean seasonal attributed ONS deaths (rate /100,000)

### Mortality

In a mean season there were an estimated 7163 respiratory (broadly defined) deaths attributable to influenza, with a mean seasonal mortality rate of 12/100,000 population (Table 1). As for hospitalisations, there was marked inter-season variability in influenza-attributable mortality rates due to respiratory disease (Fig. 1). Few influenza-attributable respiratory deaths were reported in children and adolescents <18 years of age (19/7163) (Table 4). Most deaths (74 %, 5288/7163) attributable to influenza respiratory disease were in adults aged 75+ years. The mean seasonal mortality rate attributable to influenza in those aged 75+ years was 6.5-fold higher than in those aged 65–74 years for respiratory disease (broadly defined).

**Table 4:** Mean seasonal mortality for respiratory disease^a^ attributable to influenza according to age and risk status^b^

		Influenza A		Influenza B	
Age	Risk	N (SD)	Rate (SD)	N (SD)	Rate (SD)
0–4	All	12	0	1	0
	Low	13	0	2	0
	High	3	0	0	0
5–17	All	4	0	2	0
	Low	2	0	0	0
	High	16	0	19	0
18–49	All	84	0	13	0
	Low	61	0	9	0
	High	278	1	45	0
50–64	All	402	4	27	0
	Low	145	1	16	0
	High	1316	13	71	1
65–74	All	955	19	27	1
	Low	367	7	0	0
	High	1875	38	79	2
75+	All	4776	118	512	13
	Low	5042	125	671	17
	High	4627	114	375	9
All ages^c^	All	6561 (5501)	11 (9)	602 (681)	1 (1)
	Low	3672 (3107)	6 (5)	436 (501)	1 (1)
	High	19762 (17261)	34 (29)	1395 (1569)	2 (3)

*N* Number of episodes, *SD* standard deviation, *Rate* per 100,000 population, Deaths from ONS 1996–2009

^a^broadly defined

^b^Risk group defined according the chronic conditions indicative of risk for severe influenza as per UK recommendations for influenza vaccination. These include chronic obstructive respiratory disease; cardiovascular, central nervous system, renal and liver disorders; diabetes; immunosuppressive conditions or stroke

^c^the attributions for each age group are not equal to the sum of the attributions (all ages) due to separately run models

The all age seasonal estimate for deaths due to respiratory disease (broadly defined) attributable to influenza was slightly larger than that of respiratory deaths, but was lower than deaths due to influenza-attributable cardiorespiratory disease (Table 1). Most excess influenza-attributable cardiovascular deaths were in older adults (Additional file 1: Table S4). There were more influenza-attributable respiratory (broadly defined), pneumonia and influenza and cardiorespiratory deaths (all ages) captured under the any mention criterion versus the underlying cause (Additional file 1: Table S4).

## Discussion

Using data from UK databases and laboratory surveillance, the model estimated that 28,516 hospitalisations and 7163 deaths were attributable to influenza respiratory disease in a mean season, although there was considerable between-season variability. Over the 13 seasons of the study the long term trend appeared to be one of decline, which is consistent with other UK estimates, including estimates of general practitioner consultations for influenza [4, 6]. The decline was most apparent for influenza A. The reasons for this decline are not clear, but could include the effects of repeated annual vaccination and decreasing virulence of circulating virus strains. However, declines in influenza mortality during this period have not been observed in other countries, including Spain (1998–2005), Austria (2001–2009) and the United States (1976–2007, and 1997–2009), attributed in part to increasing longevity [15–18].

The overall burden of influenza B was substantial, with hospitalisation and mortality rates approaching that of influenza A in some seasons. Influenza B-attributable hospitalisations were highest among 5–17 year olds, which lends supports to recent changes to UK influenza vaccine recommendations to extend vaccination to school children aged 5–17 years, and suggest a potential benefit for using quadrivalent influenza vaccines that contain both influenza B lineages in this age group [5].

While estimated rates of hospitalisations and death increased with age, there was a marked increase in elderly 75+ years of age, who accounted for 36 % of all influenza-attributable respiratory hospitalisations. The mean seasonal hospitalisation rate for influenza-attributable respiratory disease in 75+ year olds was more than twice that of 65–74 year olds. A modelling study of hospital admissions in England attributable to influenza between 1989 and 2001 noted that 75+ year olds accounted for 52 % of the total and 69 % of excess bed days occupancy [19]. Our findings equally emphasise the impact of influenza in this age group and the burden placed on health services. Of all influenza-associated deaths, we estimated that 74 % occurred in the 75+ year age group, although only 50 % of influenza-attributable respiratory deaths (broadly defined) in 75+ year olds occurred in hospital.

Differences in the way persons of differing ages use primary care, hospital care and welfare accommodation may bear on the interpretation of these findings. In addition, many deaths from influenza occur in persons who have not consulted a doctor, suggesting rapid death early on in the illness [20]. It is possible that some of these out-of-hospital deaths may be preventable, either through more effective vaccines for the elderly, by reducing exposure to influenza, by extending target age groups for vaccination to increase herd immunity, or by earlier access to anti-viral medication. Vaccination of children up to 17 years of age may induce herd effects, but it is not yet clear whether these effects will result in benefits for the elderly who may have little contact with young children. Increased use of post-exposure prophylaxis with anti-viral therapies in elderly persons may reduce hospitalisation and deaths in the elderly.

Several recent studies have used modelling approaches and databases that are similar to the present study [10, 21, 22]. Hardelid et al. [21], modelled all-cause mortality and found an average of approximately 12,000 influenza-attributable deaths per season for the period 1999–2010, with 79 % of these deaths occurring in 75+ years old. This is close to our estimate of about 13,000 influenza-attributable cardiorespiratory deaths over our study period. However, Pitman et al. [10] estimated a burden of up to approximately 24,000 influenza-attributable deaths per season during the earlier period 1990–2000.

Regarding the burden of hospitalisations, Cromer et al. [22], studied the seasons 2000/01 to 2007/08 using a narrower “acute respiratory illness” outcome definition and estimated that approximately 34 hospitalisations per 100,000 population occurred per season, while we estimated 49 respiratory hospitalisations and 43 cardiorespiratory hospitalisations per 100,000 population. Pitman et al. [10] found 59 cardiorespiratory hospitalisations per 100,000 population during an earlier period. Taking into consideration the differences in modelling approaches, outcome definitions and periods studied, these estimates are in reasonable agreement with one another and consistent with a long-term decline in the influenza burden [6]. The comparability with other estimates also supports the external validity of our model.

Influenza vaccination policy in the UK during the study period recommended immunisation of individuals from the age of 6 months with risk co-morbidities [12]. Our estimates show increased influenza-associated hospitalisation and death in adults with comorbid conditions, but the relative impact of comorbid conditions was lower in the elderly than in younger adults, emphasising the importance of comorbid conditions in all age groups except children. There was no apparent increase when these comorbidity factors were present in children, in whom asthma is numerically the most important risk condition (data not shown).

A strength of our study is the use of the novel outcome category ‘respiratory disease (broadly defined)’ which improved the outcome sensitivity and estimated the burden of influenza-attributable disease for all age groups consistently across the HES and ONS settings, while retaining sufficient specificity. Estimates using the respiratory disease (broadly defined) category compared well to respiratory or cardiorespiratory definitions for influenza-attributable hospitalisations. Respiratory disease (broadly defined) hospitalization estimates were somewhat larger than those for respiratory disease alone, indicating that the new definition was, as intended, more sensitive. However, influenza contributed more to excess cardiorespiratory deaths in older adults than to hospitalizations, with the result that the cardiorespiratory mortality estimate was higher. Other strengths include the long study period which covered 13 seasons, and the use of age stratification which allowed detailed estimation of the age-specific burden. Tri-mean smoothing of outcome and pathogen series reduced short-term effects such as national holidays and extreme weather conditions on viral testing and hospitalisations. We assumed that weekly occurrences of outcomes were determined by the circulation of influenza, RSV, and other causes which follow seasonal trends, that we estimated using a combination of sine and cosine terms. The inclusion of both cyclical terms (sine and cosine functions) controlled for confounders for which data are not available.

Potential limitations of our study are those related to the modelling design, which assumes firstly that the pathogen of interest is responsible for the outcomes under consideration, that hospital admission thresholds are similar nationally, and that consistency and uniformity in the quality of coding practices exists nationally. However, because we used a control outcome (hospitalisation for accidents) to detect confounding [23], the expected absence of attributions for the negative control provides support for the modelling methodology employed. We did not have access to age-stratified virology data and had to assume that patterns of influenza virus detection were similar in the adults and child populations. Finally, we extrapolated data from England, which makes up approximately 84 % of the total UK population, to the whole of the UK. While there are reasonable grounds for this approach [14], extrapolation may have resulted in over- or underestimation of the disease burden.

## Conclusions

Detailed, age-specific estimates of the total burden of influenza, including the burden in populations at higher risk for influenza complications, can contribute to cost-benefit analyses and inform decisions on vaccination and public health policy. Our results point to a substantial but highly variable seasonal influenza burden in all age groups, but particularly in those aged 75+ years. The high hospital admission mortality rate and the smaller relative proportion of overall influenza deaths happening in hospital in this age group indicates that a substantial portion of influenza-related deaths occur outside hospital, and so are not recorded as inpatients. The development of effective influenza prevention strategies in this age group is vital, as it puts major pressures on hospital services because of the increased duration of hospital admissions and difficulties surrounding discharge [19]. Influenza vaccination of all age groups to increase herd immunity and use of post-exposure prophylaxis with anti-viral medications in the elderly could potentially reduce hospitalisation and deaths due to influenza, particularly in the 75+ year population.

## Additional file

Additional file 1: Table S1. Outcomes: hospitalisations (HES) and deaths (ONS). **Table S2.** Definition of risk factors; any mention of any of these codes placed the patient in the “high-risk” category. **Table S3.** Mean number of other hospitalisations due to non-respiratory diagnoses attributable to influenza in the United Kingdom. **Table S4.** Mean number of deaths due to respiratory diagnoses attributable to influenza in the United Kingdom. (DOCX 85 kb)

## Abbreviations

CPRD: clinical practice research datalink
HES: hospital episode statistics
ICD: international classification of diseases
ONS: office of national statistics
PHE: Public Health England
RSV: respiratory syncytial virus
UK: United Kingdom
