# Supplementary material for: Modelling estimates of age-specific influenza-related hospitalisation and mortality in the United Kingdom
Source: BMC Public Health. 2016 Jun 8;16:481. doi: 10.1186/s12889-016-3128-4 (PMC4898386; doi:10.1186/s12889-016-3128-4)
Supplement: Additional file 1: Table S1. — Outcomes: hospitalisations (HES) and deaths (ONS). Table S2. Definition of risk factors; any mention of any of these codes placed the patient in the “high-risk” category. Table S3. Mean number of other hospitalisations due to non-respiratory diagnoses attributable to influenza in the United Kingdom. Table S4. Mean number of deaths due to respiratory diagnoses attributable to influenza in the United Kingdom. (DOCX 85 kb) [file 12889_2016_3128_MOESM1_ESM.docx]

**Modelling estimates of age-specific influenza-related hospitalisation and mortality in the United Kingdom**

Gonçalo Matias, Robert J Taylor, François Haguinet, Cynthia Schuck-Paim, Roger L Lustig, Douglas M Fleming

**Supplementary data**

**Description of databases**

*Hospital Episode Statistics: HES*

Each HES record contained data on one hospitalisation “episode,” defined as a period of hospitalized care under a single consulting physician. Some patients are transferred between consulting physicians before being discharged; all continuous episodes make up one “spell” of hospitalisation. HES records contain information on discharge diagnosis, admission date, patient age, type of patient (admitted or day patient), number of days spent in the hospital, status at discharge (alive or dead) and more. Each episode had up to 20 ICD10-coded discharge diagnoses listed. The first listed diagnosis in any episode is the primary reason for the hospitalisation; for spells with multiple episodes, we took the first listed diagnosis in the last spell to be the underlying diagnosis. We only included patients who came to the hospital on an emergency basis, as opposed to scheduled stays. In 2009-10 there were 5,177,887 admissions through the emergency department of which 1,067,134 were admissions for cardiovascular or respiratory diseases. ICD-9-coded data were not used as they encompassed only two study years and because of difficulties mapping codes from one ICD version to another.

*Office of National Statistics: ONS*

The UK ONS maintains a registry of all deaths in UK, coded using ICD classification of cause. About 550,000 deaths are recorded each year. The underlying cause is the primary reason for a person’s death; other contributing causes are recorded as well. We requested and received all records for decedents in England whose stated causes of death included any ICD9/10 code of interest to the study. Data were ICD9-coded through December 2000 and ICD10-coded thereafter. Adjustments for the change from ICD-9 to ICD-10 were made (as recommended by ONS [1]) to produce time series that were not substantially different before and after the version change.

**Reference**

1. Brock A, Griffiths C, Rooney C: The impact of introducing ICD-10 on analysis of respiratory mortality trends in England and Wales. *Health Stat Q* 2006:9–17.

**Supplementary Table 1:** Outcomes: hospitalisations (HES)* and deaths (ONS)**

| **Outcome** | **ICD10 codes** |
| --- | --- |
| **Respiratory Diseases** | |
| Cardiorespiratory | I00-99,J00-99 |
| Respiratory Disease Broadly Defined: Resp Dis + Symptoms + Sepsis | J00-99, R05-06, B34, A40-41, P36 |
| Respiratory Disease | J00-99 |
| Pneumonia & influenza | J09-18 |
| **Non-Respiratory Disease** | |
| Cardiovascular disease | I00-99 |
| Renal Disease | N00-28 |
| CNS-related outcomes | A80-89, G00-04, G93.7, |
| Diabetes | E10-E14 |
| Accidents | V00-99,X00-99,Y00-99 |

*Only unscheduled, “emergency” hospitalisations were included

**Registered (underlying) cause of death

**Supplementary Table 2:** Definition of risk factors; any mention of any of these codes placed the patient in the “high-risk” category

| **Risk Factor** | **ICD 10** | **ICD 9** |
| --- | --- | --- |
| COPD | J40-J47 | 490-496 |
| Cardiovascular disorders | I00-I52 [except I01-04, I10, I30, I32-33, I40, I46, I49.1, I49.4] | 393-400, 419-420, 423-429 [except 427.6] |
| Kidney disorders | N00-N29 [except N00, N10, N17, N20-23] | 508-587 [except 580,584] |
| Diabetes | E10-E14 | 250 |
| Immunosuppression | B20-24, O98.7, C00-C99, D37-D48, Z21 | 042,140-208, 230-238 |
| Liver disorders | K70-K77 | 570-573 |
| Stroke | I60-I69 | 430-438 |
| CNS disorders | Q00-Q07, G10-G39, G45-46, G70-G99 | 330-349 [except 345, 346], 350-359 |

**Supplementary Table** **3:**  Mean number of other hospitalisations due to non-respiratory diagnoses attributable to influenza in the United Kingdom

| **Outcome** | **Age** | **Influenza A** | **Influenza B** |
| --- | --- | --- | --- |
| Central nervous system | 0-4 | 1 | 0 |
| (Primary diagnosis) | 5-17 | 8 | 10 |
|  | 18-49 | 0 | 2 |
|  | 50-64 | 6 | 0 |
|  | 65+ | 2 | 0 |
|  | All ages | 0 | 5 |
| Cardiovascular disease | 0-4 | 0 | 0 |
| (Primary diagnosis) | 5-17 | 0 | 45 |
|  | 18-49 | 0 | 0 |
|  | 50-64 | 0 | 0 |
|  | 65+ | 110 | 0 |
|  | All ages | 0 | 0 |
| Diabetes | 0-4 | 8 | 2 |
| (Primary diagnosis) | 5-17 | 0 | 19 |
|  | 18-49 | 7 | 22 |
|  | 50-64 | 23 | 11 |
|  | 65+ | 9 | 7 |
|  | All ages | 14 | 60 |
| Renal disease | 0-4 | 0 | 2 |
| (Primary diagnosis) | 5-17 | 0 | 11 |
|  | 18-49 | 0 | 23 |
|  | 50-64 | 9 | 6 |
|  | 65+ | 44 | 0 |
|  | All ages | 0 | 35 |

**Supplementary Table** **4**  Mean number of deaths due to respiratory diagnoses attributable to influenza in the United Kingdom

| **Outcome** | **Age** | **Influenza A** | **Influenza B** |
| --- | --- | --- | --- |
| Respiratory disease broadly defined | 0-4 | 17 | 2 |
| (Any mention) | 5-17 | 9 | 7 |
|  | 18-49 | 151 | 40 |
|  | 50-64 | 643 | 60 |
|  | 65+ | 9551 | 1085 |
|  | All ages | 10642 | 1226 |
| Cardiorespiratory | 0-4 | 17 | 1 |
| (Any mention) | 5-17 | 8 | 7 |
|  | 18-49 | 160 | 30 |
|  | 50-64 | 826 | 55 |
|  | 65+ | 11691 | 1327 |
|  | All ages | 13030 | 1460 |
| Pneumonia & influenza | 0-4 | 9 | 2 |
| (Any mention) | 5-17 | 7 | 3 |
|  | 18-49 | 116 | 21 |
|  | 50-64 | 396 | 36 |
|  | 65+ | 7168 | 866 |
|  | All ages | 7901 | 953 |
